# Data-Independent Acquisition Parallel Accumulation–Serial Fragmentation (diaPASEF) Analysis of the Separated Zebrafish Lens Improves Identifications

**DOI:** 10.1021/jasms.5c00087

**Published:** 2025-06-09

**Authors:** Sarah R. Zelle, W. Hayes McDonald, Kristie L. Rose, Hassane S. Mchaourab, Kevin L. Schey

**Affiliations:** † Chemical and Physical Biology Program, 5718 Vanderbilt University, Nashville, Tennessee 37212, United States; ‡ Department of Biochemistry, 5718 Vanderbilt University, Nashville, Tennessee 37212, United States; § Department of Molecular Physiology and Biophysics, Vanderbilt University, Nashville, Tennessee 37232, United States

**Keywords:** DIA, diaPASEF, proteomics, zebrafish, lens, aging

## Abstract

Ocular lens fiber cells degrade their organelles during differentiation to prevent light scattering. Organelle degradation occurs continuously throughout an individual’s lifespan, creating a spatial gradient of young cortical fiber cells in the lens periphery to older nuclear fiber cells in the center of the lens. Therefore, separation of cortical and nuclear regions enables examination of protein aging. Previously, the human lens cortex and nucleus have been studied using data-independent acquisition (DIA) proteomics, allowing for the identification of low-abundance protein groups. In this study, we employed data-independent acquisition parallel accumulation–serial fragmentation (diaPASEF) proteomics on a timsTOF HT instrument to study the zebrafish lens proteome and compared results to a standard DIA method employed on an Orbitrap Exploris 480 mass spectrometer. Using the additional ion mobility gas phase separation of diaPASEF, peptide and protein group identifications increased by over 200% relative to an Orbitrap DIA method in the zebrafish lens. With diaPASEF, we identified 13,721 and 11,996 unique peptides in the cortex and nucleus of the zebrafish lens, respectively, which correspond to 1,537 and 1,389 protein groups. Thus, separation of the zebrafish lens into cortical and nuclear regions followed by diaPASEF analysis produced the most comprehensive zebrafish lens proteomic data set to date.

## Graphical abstract

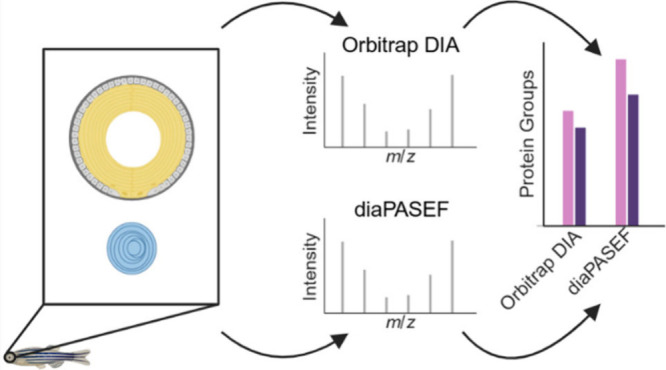

## Introduction

The lens is a transparent organ that focuses light onto the retina. With age, the lens becomes opacified and scatters light, a condition known as cataract, the leading cause of blindness worldwide.^1–3^ Cellular organelles act as potential sources of light scattering, so during lens fiber cell differentiation, organelles are degraded.^1^ As we age, continual lens fiber cell differentiation creates a spatial gradient of young cortical fiber cells to old nuclear fiber cells (Figure 1). Due to the loss of organelles during differentiation, there is no protein turnover in nuclear lens fiber cells, and proteins synthesized at birth remain in the nucleus for the lifespan of an individual.^2–7^ Lens fiber cell differentiation thus creates a continuum of regions with distinct proteomes, necessitating the separation of lens regions to examine age-related proteome changes. Conventionally, the lens is separated into cortical and nuclear regions for spatiotemporal proteomic analyses.^8,9^

**Figure 1:**
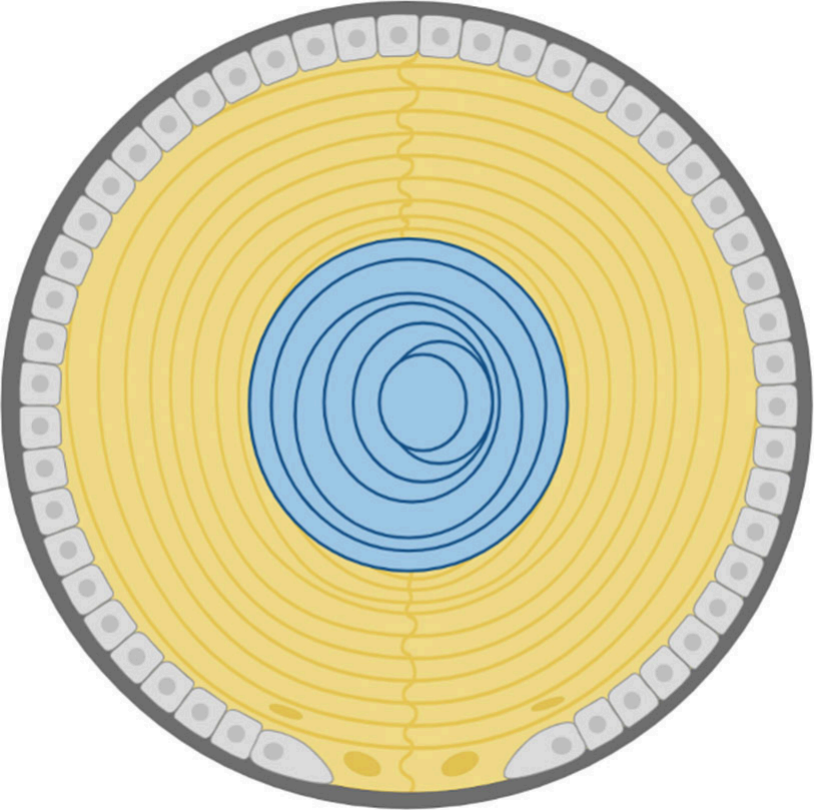
**Diagram of the adult zebrafish lens.** Epithelial cells (light gray) differentiate into lens fiber cells. The outer layer of lens fiber cells is the cortex (yellow), while the inner layer is the nucleus (blue). The lens is enclosed in the capsule, which is comprised of basement membrane (dark gray). Figure created using Biorender.com.

Abundance-based data-dependent acquisition (DDA) mass spectrometry proteomics and labeling methods such as tandem mass tags have been used to quantify proteins and post-translational modifications in the lens.^10–15^ However, due to the high concentration of crystallins, up to 300 mg/mL in the human lens, these methods are challenged to identify and quantify low-abundance proteins.^16^ Thus, a new data-independent acquisition (DIA) mass spectrometry proteomics method was developed to study the human lens proteome.^17,18^ Using a 95 min liquid chromatography gradient combined with a library-free DIA method on an Orbitrap Exploris 480 instrument to interrogate the human lens proteome, Cantrell and Schey identified 39,292 peptides, corresponding to 4,476 protein groups in the lens cortex. In contrast, an Orbitrap DDA method on the same instrument identified 9,448 peptides and 1,739 corresponding protein groups in the lens cortex. These results show that by using Orbitrap DIA there was an almost 250% increase in peptide identifications and an almost 400% increase in protein group identifications. Fewer peptides and protein groups were identified in the lens nucleus region relative to the cortex, but the Orbitrap DIA method also identified more peptides and protein groups relative to the Orbitrap DDA method. Data-independent acquisition parallel accumulation–serial fragmentation (diaPASEF) mass spectrometry proteomics takes advantage of an additional ion mobility gas phase separation device and high-speed mass analysis to increase the number of peptide and protein group identifications.^19^ Here, we pioneer the use of diaPASEF mass spectrometry proteomics to study the zebrafish lens and compare our results to data acquired using the previously reported Orbitrap DIA method.

Zebrafish have several attributes that make them ideal model organisms for studying the lens proteome. For instance, zebrafish undergo a similar lens organogenesis process and have an analogous lens structure to humans.^20,21^ Previously, several zebrafish lens proteomic studies fractionated and used matrix-assisted laser desorption–ionization time-of-flight DDA mass spectrometry to determine the expression and ratio of crystallins during development and aging.^22–24^ Posner et al. also applied parallel reaction monitoring mass spectrometry to examine the presence of α-crystallins during zebrafish development.^25^ Finally, Wu et al. utilized isobaric labeling mass spectrometry to quantify proteomic changes in the lenses of γ-crystallin zebrafish mutants.^26^ However, the zebrafish lens has traditionally been analyzed intact due to its small size. Thus, for the first time, the nuclear and cortical regions of the zebrafish lens were separated for proteomic analysis, allowing for the examination of lens fiber cell aging. By utilizing diaPASEF proteomics, we collected the most comprehensive proteomic data set of the zebrafish lens to date.

## Materials and Methods

All reagents were purchased from Sigma-Aldrich (St. Louis, MO), and S-Trap micro columns were acquired from Protifi (Farmingdale, NY). Mass spectrometry grade trypsin and other remaining materials were purchased from Fisher Scientific (Waltham, MA) unless noted otherwise.

### Zebrafish Maintenance and Breeding

AB-WT strain zebrafish (*Danio rerio*) embryos were raised to 10 months of age. Zebrafish were kept in 30 mg/L instant ocean in deionized water at 28.5 °C and on a 14:10 h light/dark cycle. The Vanderbilt University Institutional Animal Care and Use Committee authorized all zebrafish procedures.

### Dissection and Separation of the Lens

Fresh lenses from three zebrafish from the same tank were dissected from the eye and placed in cold phosphate-buffered saline.^27^ To separate the lens cortical and nuclear regions, 5 μL of cold 100 mM Tris pH 7.8, 1.5 mM ethylenediaminetetraacetic acid (EDTA) buffer was first placed in the neck of Biotik 20 μL xTIP4 pipet tips (catalog number 63300010). Lenses were then gently dried on a Kimwipe using tweezers and put into the buffer droplet in the pipet tip. The tips were spun at 5,000*g* for 30 s at 4 °C in Gl Sciences centrifuge adapters inserted into 1.5 mL microcentrifuge tubes. The centrifugation process shears off the outer (cortical) region of the lens into the eluted solution, while the center (nucleus) of the lens remains intact and stuck in the tip. The tips were then cut using a razor blade at a point just below where the lens nucleus was held. Tweezers were used to free the nucleus by pushing it toward the larger end of the tips and spinning again to elute the intact nucleus into the buffer containing the separated cortex region. To wash the tips of any lingering cortical tissue, 10 μL of cold buffer was added and the tips were spun again. The buffer containing the cortical region was then transferred to another tube, leaving the intact nucleus inside the initial tube. The exterior of the nucleus was then washed by suspending it in 10 μL of cold buffer to remove any remaining attached cortical fiber cells. The wash buffer was then transferred to the cortical fraction and the separated regions were then snap-frozen for storage at −80 °C.

### Preparation of Samples for Mass Spectrometry Proteomic Analysis

Separated tissue samples were thawed and 50 μL of 100 mM Tris pH 7.8, 1.5 mM EDTA, and 5% sodium dodecyl sulfate (SDS) buffer was added to the nuclear samples, while 25 μL of 100 mM Tris pH 7.8, 1.5 mM EDTA, and 10% SDS buffer was added to the cortical samples. All samples were homogenized for 15 s using a hand-held Fisherbrand pellet pestle and spun down at 18,213*g* for 5 min to remove SDS-insoluble material. Protein concentration in the supernatant was measured using a bicinchoninic assay. Cysteines in 10 μg of protein were reduced for 1 h at 56 °C using 10 mM of dithiothreitol and alkylated for 30 min in the dark at room temperature using 50 mM of iodoacetamide. Proteins were acidified by adding a 1:10 ratio of sample to 27.5% phosphoric acid and then precipitated onto an S-Trap micro spin column by adding a 6:1 ratio of 100 mM triethylammonium bicarbonate (TEAB) in 90% methanol to sample and centrifuged at 4,000*g* for 30 s. Samples were washed three times with 50% chloroform/50% methanol, followed by four additional washes with 100 mM TEAB in 90% methanol. Proteins were digested by adding 50 mM TEAB with a 1:10 ratio of 10 μg sample to 1 μg of mass spectrometry grade trypsin and incubated for 2 h at 47 °C. Digested peptides were eluted from S-Traps sequentially by adding 50 mM TEAB, 0.2% formic acid (FA), 50% acetonitrile, and 0.2% FA solutions and centrifuging at 4,000*g* for 1 min each. Eluted peptides were dried with a vacuum concentrator and stored at −80 °C.

### Data Acquisition

Peptides were resuspended in 0.2% FA to a concentration of 200 ng/μL. Peptide concentrations were estimated using a Nanodrop 2000 Spectrophotometer (Thermo Scientific), and samples were adjusted to 50 ng/μL. 0.015% n-dodecyl-β-d-maltoside was added to diaPASEF samples. DiaPASEF data were acquired on 150 ng of peptides using a 30-min aqueous-to-organic gradient method delivered via a nanoELUTE2 on a PepSep column with 75-μm internal diameter, 25-cm length, and 1.5-μm particle size coupled to a timsTOF HT instrument (Bruker) using a 20-μm CaptiveSpray emitter. diaPASEF data were collected in 12 PASEF ramps from 0.75 to 1.3 1/*k*_0_ covering 350 to 1250 *m*/*z* via variable windows ranging in size from 12.27 to 122.81 Th with 50 ms accumulation time. Orbitrap DIA data were acquired on 150 ng of peptides using a 95 min aqueous-to-organic gradient method delivered via a Dionex Ultimate 3000 UHPLC on a 360 μm outer diameter × 100 μm internal diameter column packed with 20 cm of 3 μm C18 reverse phase material (Jupiter, Phenomenex) coupled to an Orbitrap Exploris 480 instrument (Thermo Scientific). Precursor spectra from 385 to 1025 *m*/*z* were collected after thirty 20 *m*/*z* windows of DIA MS/MS spectra from 400 to 1000 *m*/*z* or thirty-one 20 *m*/*z* windows of DIA MS/MS spectra from 390 to 1010 *m*/*z*.^17,18^

### Data Analysis and Visualization

Database searching for all files was performed using DIA-NN 1.9 with the default library-free settings and the reannotate and contaminants options selected.^28,29^ A UniProt Swiss-Prot and TrEMBL zebrafish FASTA database (UP000000437, downloaded 9/10/2024, 25,999 entries with only one protein per gene) was used. Match between runs was turned on and MS1 and MS2 mass accuracy were fixed at 10 ppm. The default fixed N-terminal methionine excision and cysteine carbamidomethylation modifications and no variable modifications were selected. Searching was performed on an Intel Core i7-10700 CPU at 2.90 GHz.

Data were analyzed and visualized using custom R scripts on noncontaminant peptides having <1% q-value and at least 2 unique peptides per protein group. DIA-NN normalization was rejected, and median normalization was performed separately on files for each instrument ().^17,18^ Abundances were calculated using the diann_maxlfq function from the diann R package.^28^ Figures were generated in R using ggplot2, ggvenn, and EnhancedVolcano packages. The prcomp package was used to perform principal component analysis (PCA) on untransformed data for all protein groups without missing values in the diaPASEF and Orbitrap DIA data sets separately. A Welch’s *t*-test between cortical and nuclear samples was performed on all proteins. Results were plotted using a volcano plot with significance cut-offs at 0.05 p-value and ± 1.5 log_2_ fold change. PANTHER was used for overrepresentation analyses of all proteins with a significant p-value <0.05 as determined via Welch’s *t*-test using the 06/17/2024 GO database.^30^ Statistically significant cortical and nuclear proteins from the diaPASEF and Orbitrap DIA data sets were analyzed separately using a Fisher’s exact test with false discovery rate (FDR) calculated. All identified proteins from both data acquisitions comprised the reference proteome, and only parent GO terms with FDR p-value <0.05 were considered. Data are available via ProteomeXchange with identifier PXD059560 and R scripts utilized for analysis are available on request.

## Results and Discussion

Biotik 20 μL xTIP4 pipet tips were found to be the appropriate size to roughly separate the adult zebrafish lens cortex and nucleus regions. The pipet tip separation method is successful because the lens nucleus is harder than the lens cortex.^31,32^ Thus, lenses of different sizes can be separated based on the size of the nucleus. However, this technique can only be used on fresh adult zebrafish lenses, as lenses from zebrafish that have not yet reached sexual maturity were too small and passed through the pipet tip intact.

Due to the additional gas phase separation and the faster instrument acquisition speed, the diaPASEF method was predicted to increase the number of peptide and corresponding protein group identifications relative to Orbitrap DIA in the zebrafish lens cortex and nucleus.^33,34^ Results revealed that among three biological replicates, an average of 3,584 unique peptides were identified using an Orbitrap DIA method in the zebrafish cortex, and an average of 3,103 unique peptides were identified in the zebrafish nucleus. However, by using diaPASEF these numbers increased to an average of 13,721 unique peptides identified in the zebrafish lens cortex, and 11,996 unique peptides in the nucleus, representing about a 260% average increase in peptide identifications in both regions (Figure 2A). In the Orbitrap DIA data sets, these peptides can be assigned, on average, to 511 unique protein groups in the cortex, and 432 unique protein groups in the nucleus. In the diaPASEF data sets, these numbers increased to an average of 1,537 unique protein groups in the cortex, and an average of 1,389 unique protein groups in the nucleus (Figure 2B). These identifications represent about a 330% increase in cortical protein group identifications and about a 310% increase in nuclear protein group identifications using diaPASEF. However, the Orbitrap DIA method used in this comparison did not employ a high field asymmetric waveform ion mobility spectrometry (FAIMS) device. FAIMS has been shown to increase proteome coverage in Orbitrap DIA methods.^35,36^

**Figure 2:**
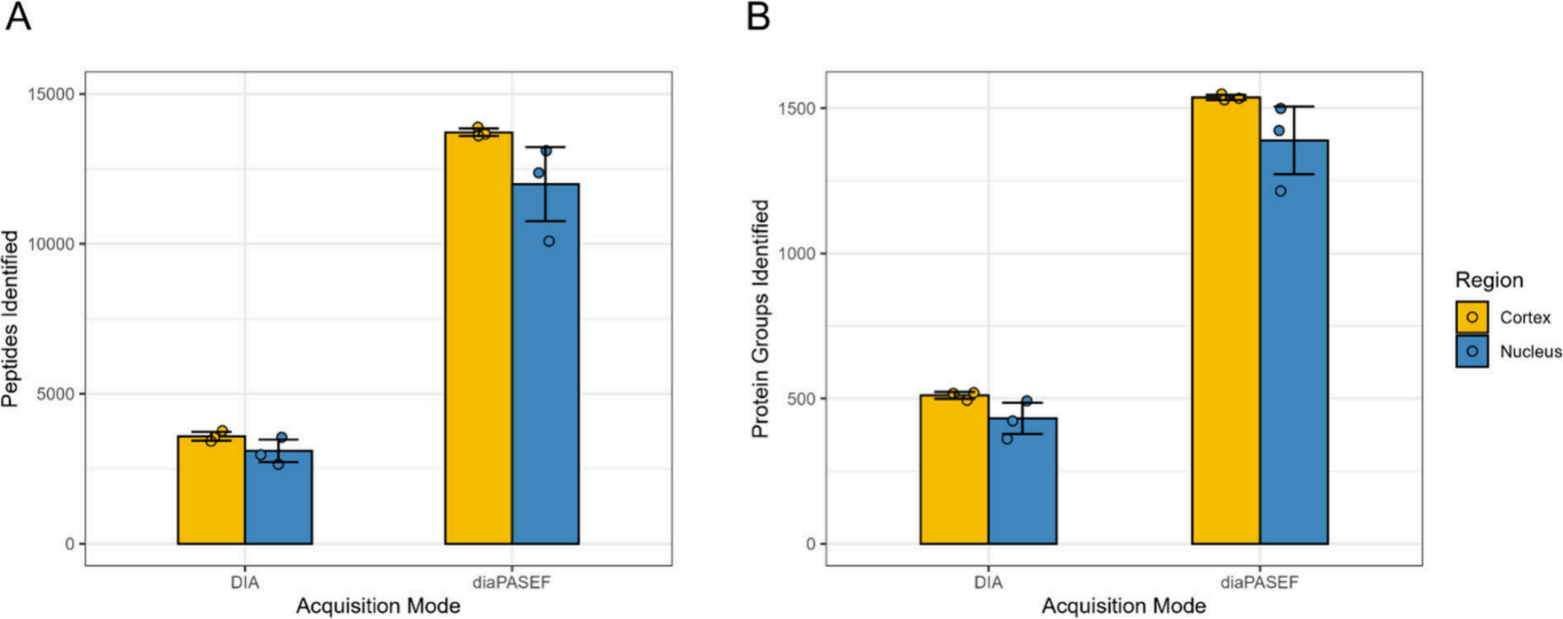
**Bar plots of Orbitrap DIA and diaPASEF results for three biological replicates displayed by acquisition mode and region. A)** number of peptides identified and **B)** number of corresponding protein groups identified. A total of 16,718 unique peptides and 1,674 protein groups were identified throughout all sample types and data acquisition strategies.

We next compared the overlap between identifications on the peptide and protein group level in the zebrafish cortex and nucleus for both data acquisition methods. On the peptide level, about 20% of all identifications in the lens cortex and nucleus were detected using both Orbitrap DIA and diaPASEF. However, over 70% of all identified peptides were found exclusively in the diaPASEF data sets in both regions, suggesting ion mobility is needed to identify these additional peptides (Figure 3A) within a shorter acquisition time. At the protein group level, there was more overlap, with more than 30% of all protein groups identified in both regions using both acquisition methods. Over 60% of all protein groups were found only in the diaPASEF data sets for both regions, suggesting that diaPASEF gives a more complete protein coverage in the zebrafish lens (Figure 3B). The diaPASEF data sets also had, compared to the Orbitrap DIA data sets, a lower median MS2-based peptide intensity percent coefficient of variation (% CV) and proportion of peptides with a lower % CV (). Additionally, there is a notable overlap between the cortex and nucleus regions in identifications on both the peptide and protein group levels in both data acquisition methods (). Differences in peptide and protein group identifications were likely due to protein modification, truncation, degradation, or differences in protein synthesis pathways, processes associated with fiber cell aging.^8,14,37,38^ A high degree of similarity was also found between replicates (). However, unique peptides and protein groups found in only one replicate are likely a result of low-abundance peptides being less reproducible and of the biological variance found in AB-WT zebrafish.^39,40^ These results demonstrate that diaPASEF facilitates the quantification of more unique peptides and protein groups in the zebrafish lens compared to the Orbitrap DIA method, allowing for deeper investigation into lens biology.

**Figure 3:**
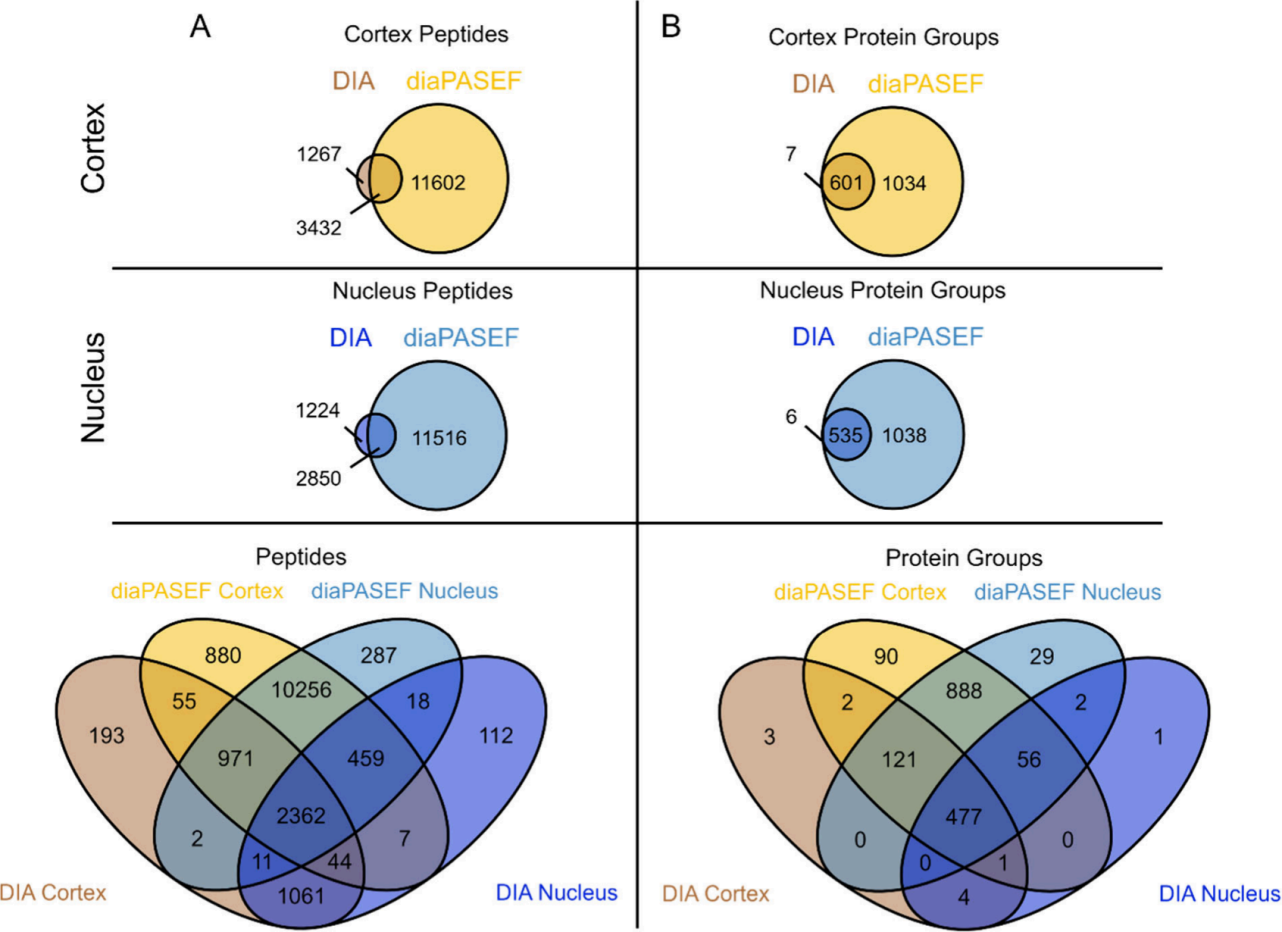
**Venn diagrams showing the overlap between acquisition methods for A)** peptides and **B)** corresponding protein groups identified in the zebrafish lens cortex and nucleus. All peptides and protein groups found in at least one biological replicate were considered, with duplicates removed.

Next, we validated the separation of the zebrafish lens cortex and nucleus regions by examining the observed proteins in the diaPASEF data sets. A PCA plot using untransformed MaxLFQ quantities for 1,103 protein groups found in all diaPASEF samples showed that the samples separated into two distinct clusters: cortical and nuclear (Figure 4A). The same pattern was observed in the Orbitrap DIA data ().

**Figure 4:**
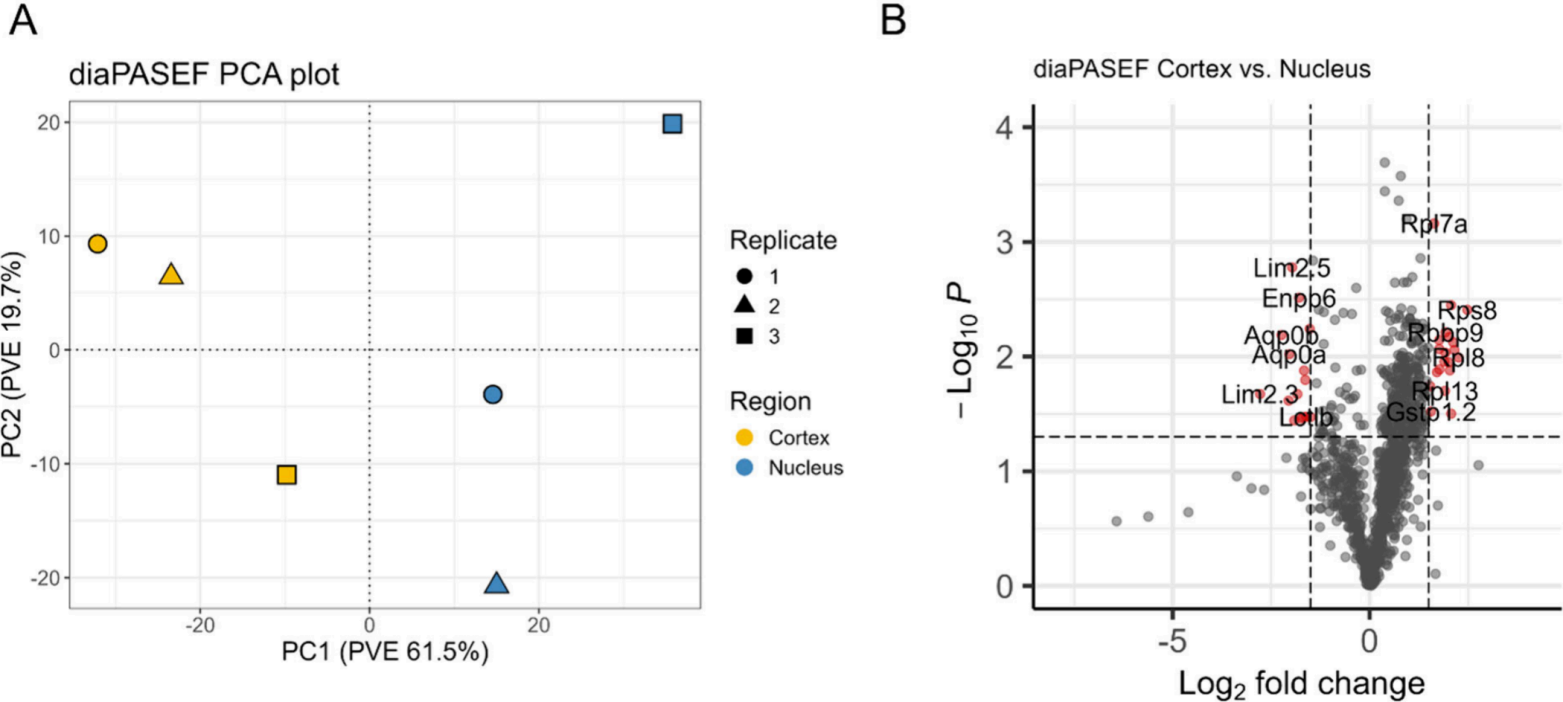
**Analysis of diaPASEF data. A)** PCA plot separated by replicate and region (*n* = 1,103). **B)** Volcano plot of proteins in cortex vs nucleus samples. Significant protein groups are shown in red with cut-offs of log_2_FC >1.5 or log_2_FC <−1.5 and p-value <0.05 as calculated from Welch’s *t*-test.

Additionally, protein abundances were compared between the nuclear and cortical diaPASEF data sets via a volcano plot to identify differentially expressed proteins. No imputation was performed and only proteins found in all three replicates were considered (Figure 4B). The results revealed that several ribosomal proteins such as Rps8, Rpl8, Rpl13, and Zgc:171772 were differentially expressed in the cortex relative to the nucleus. The differential expression of ribosomal proteins is consistent with known biology in that protein translation occurs in outer regions of the cortex and that organelles are degraded during fiber cell differentiation, leading to a loss of ribosomal proteins in older fiber cells.^1^ Our results also show that Gstp1.2, an enzyme involved in the conjugation of glutathione, is differentially expressed in the cortex. Glutathione is a major lens antioxidant important in preventing cataract formation.^41,42^

In the lens nucleus, structural membrane proteins, such as Lim 2.5, Aqp0a, and Aqp0b were differentially expressed.^43^ In particular, Aqp0a, Aqp0b, Gja8b, and Slc20a1b are all part of the lens microcirculation system that imports nutrients and exports waste products to and from the lens nucleus.^44–47^ Only one γ-crystallin protein, Si:dkey-57a22.15, was differentially expressed in the lens nucleus. However, upon further examination, Crygm2d7, Crygm2d13, Crygm2d12, Crygmxl2, Crygm2e, and Crygm2f all had Log_2_FC values that met the significance cutoff but had large p-values. These proteins would likely have been identified as significantly expressed in the nucleus if more than three biological replicates were analyzed. Metabolically active proteins, such as Enpp6 and Lctlb were also differentially expressed in the lens nucleus, suggesting that some metabolically active proteins are retained. Lctlb has also been shown to be involved in lens suture and cataract formation in mice.^48,49^ These results showed that overall, more lens-specific proteins were found in the nuclear region, indicative of the successful separation of the lens cortex and nucleus.

A full list of significant proteins, as visualized in the volcano plot, is provided in . Similar trends were also observed in the Orbitrap DIA method volcano plot (). Significant proteins in the Orbitrap DIA method volcano plot are shown in . Only 5 significant proteins were found in both data sets, Rpl13, Gstp1.2, Si:dkey-164f24.2, Lctlb, and Aqp0b. Of the 24 remaining nonoverlapping significant proteins identified in the Orbitrap DIA data, 23 were not significant in the diaPASEF data, and 1 was detected exclusively in the Orbitrap DIA data. However, 22 out of these 23 proteins met the p-value cutoff but did not meet the log_2_FC cutoff. Therefore, in the diaPASEF data there was a statistically significant but small difference between the expression of these proteins in the cortex and the nucleus. This result can be due to differences such as duty cycle, dynamic range, and sensitivity between the instruments and methods.

Finally, we performed an overrepresentation analysis for significant proteins identified via Welch’s *t*-test for both diaPASEF cortical and nuclear data sets using PANTHER.^26^ In the cortex diaPASEF data set, there is an overrepresentation of ribosomal and translation-associated gene ontologies (Table 1). Due to the lack of organelle-associated terms in the GO cellular component overrepresentation analysis, the cortex samples are likely mostly comprised of lens fiber cells undergoing or having completed differentiation.

**Table 1:** Overrepresentation Analysis of Statistically Significant Cortical diaPASEF Proteins (p-value <0.05 and log_2_FC >1.5)

GO Term	Annotation	No. in Set	Fold Enrichment	False Discovery Rate
**GO Biological Process**				
GO:0006412	Translation	15	7.94	8.55 × 10^–9^

**GO Molecular Function**				
GO:0003735	Structural constitutent of ribosome	15	16.4	0.344

**GO Cellular Component**				
GO:0022625	Cytosolic large ribosomal subunit	12	24.32	1.54 × 10^–13^

Conversely, the diaPASEF nucleus data set exhibits an overrepresentation of water transmembrane transporter activity, catalase activity, cell-junction, and membrane gene ontologies (Table 2).

**Table 2:** Overrepresentation Analysis of Statistically Significant Nuclear diaPASEF Proteins (p-value <0.05 and log_2_FC <−1.5)

GO Term	Annotation	No. in Set	Fold Enrichment	False Discovery Rate
**GO Biological Process**				
No statistically significant results				

**GO Molecular Function**				
GO:0005372	Water transmembrane transporter activity	2	>100	0.052
GO:0004096	Catalase activity	2	>100	0.0346

**GO Cellular Component**				
GO:0005911	Cell–cell junction	4	11.36	0.0231
GO:0005886	Plasma membrane	10	4.33	0.00295
GO:0005737	Cytoplasm	3	0.28	0.00807

These gene ontology results further validate the successful separation of the younger lens cortex from the older lens nucleus. Overrepresentation analysis results for the Orbitrap DIA cortex data set are shown in . No overrepresentation analysis was performed on the Orbitrap DIA nucleus data set, due to the low number of significantly expressed proteins.

## Conclusion

We have shown that a 30-min liquid chromatography diaPASEF method on a timsTOF instrument identifies more peptide and protein groups in the lens than a 95-min liquid chromatography DIA method on an Orbitrap instrument due to a shorter acquisition time and the addition of ion mobility. Thus, using diaPASEF, we have collected the most comprehensive zebrafish lens proteome data set to date. Through the analysis of the diaPASEF data sets, we have also demonstrated the first instance of successful separation of the zebrafish lens cortex and nucleus regions. DiaPASEF is a methodology that will allow further probing of the lens proteome using model organisms to more thoroughly understand lens biology.

## Supplementary Material





## Notes

Median normalization boxplots, peptide intensity percent CV per sample, Venn diagrams comparing overlap between regions, Venn diagrams comparing overlap between replicates, Orbitrap DIA PCA and volcano plots, tables of significant proteins identified in diaPASEF and Orbitrap DIA data sets, and significant GO terms for Orbitrap DIA cortex data set (PDF)
MaxLFQ results for diaPASEF and Orbitrap DIA (XLSX)
