# Supplementary material for: Data-Independent Acquisition Parallel Accumulation–Serial Fragmentation (diaPASEF) Analysis of the Separated Zebrafish Lens Improves Identifications
Source: J Am Soc Mass Spectrom. 2025 Jun 9;36(7):1503–9. doi: 10.1021/jasms.5c00087 (PMC12232379; doi:10.1021/jasms.5c00087)
Supplement: Supplementary file 1 [file js5c00087_si_001.pdf]

Supporting Information

Data-independent acquisition parallel accumulation-serial fragmentation (diaPASEF) analysis of the separated zebrafish lens improves identifications

Sarah R. Zelle[1], W. Hayes McDonald[2], Kristie L. Rose[2], Hassane S. Mchaourab[3], Kevin L. Schey[2*]

[1]Chemical and Physical Biology Program, Vanderbilt University, Nashville, Tennessee 37212, United States.

[2]Department of Biochemistry, Vanderbilt University, Nashville, Tennessee 37212, United States.

[3]Department of Molecular Physiology and Biophysics, Vanderbilt University, Nashville, Tennessee 37232, United States.

*** Corresponding Author:** k.schey@vanderbilt.edu

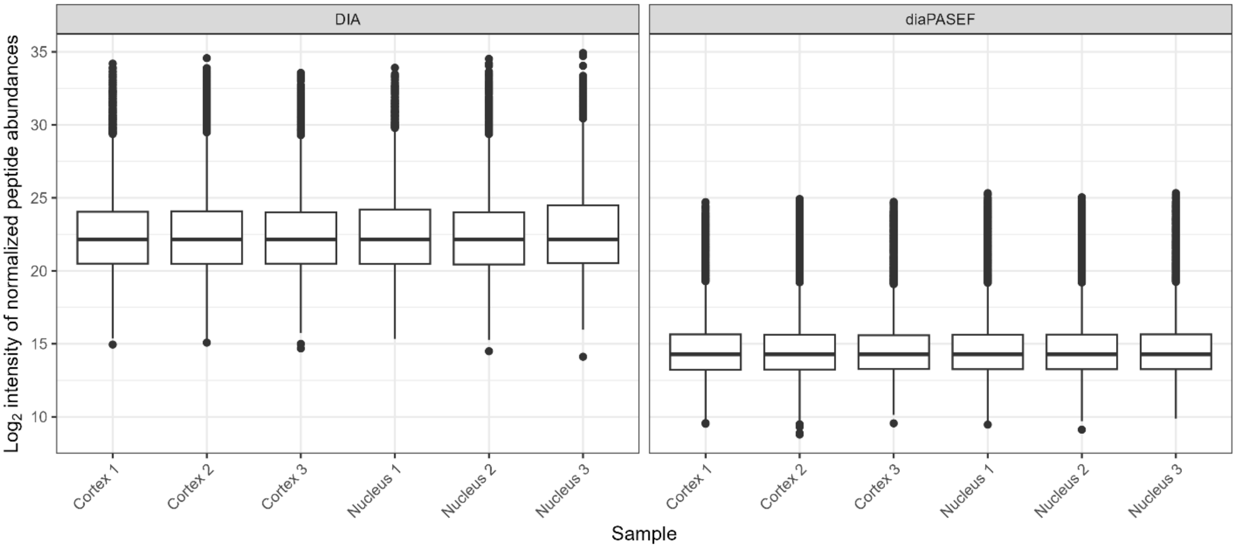

Supplemental Figure 1. Log$_2$ quantity of peptide MS2 intensities after median normalization on DIA data collected on a Thermo Exploris 480 instrument and diaPASEF data collected on a Bruker timsTOF HT instrument.

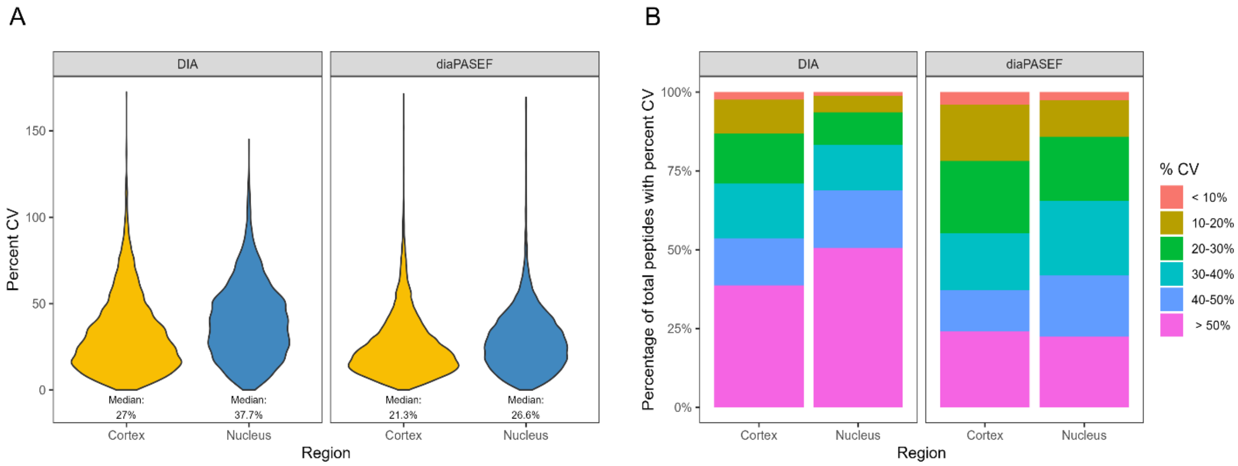

Supplemental Figure 2. Peptide intensity % CV results for the separated zebrafish lens cortex and nucleus. All peptides with < 1% q-value and at least 2 unique peptides per protein group were considered. Results were not separated by biological replicate or filtered to include only unique peptides. In total, 9,540 peptides were analyzed for the orbitrap DIA cortex samples, 8,087 peptides were analyzed for the orbitrap DIA nucleus samples, 40,211 peptides were analyzed for the diaPASEF cortex samples, and 33,492 peptides were analyzed for the diaPASEF nucleus samples. **A)** Violin plot of % CV for cortical and nuclear peptides for data collected using both DIA on a Thermo Exploris 480 and diaPASEF on a Bruker timsTOF HT. **B)** Bar plot of % CVs for cortical and nuclear peptides broken down by percentage of peptides within a specific range of % CV.

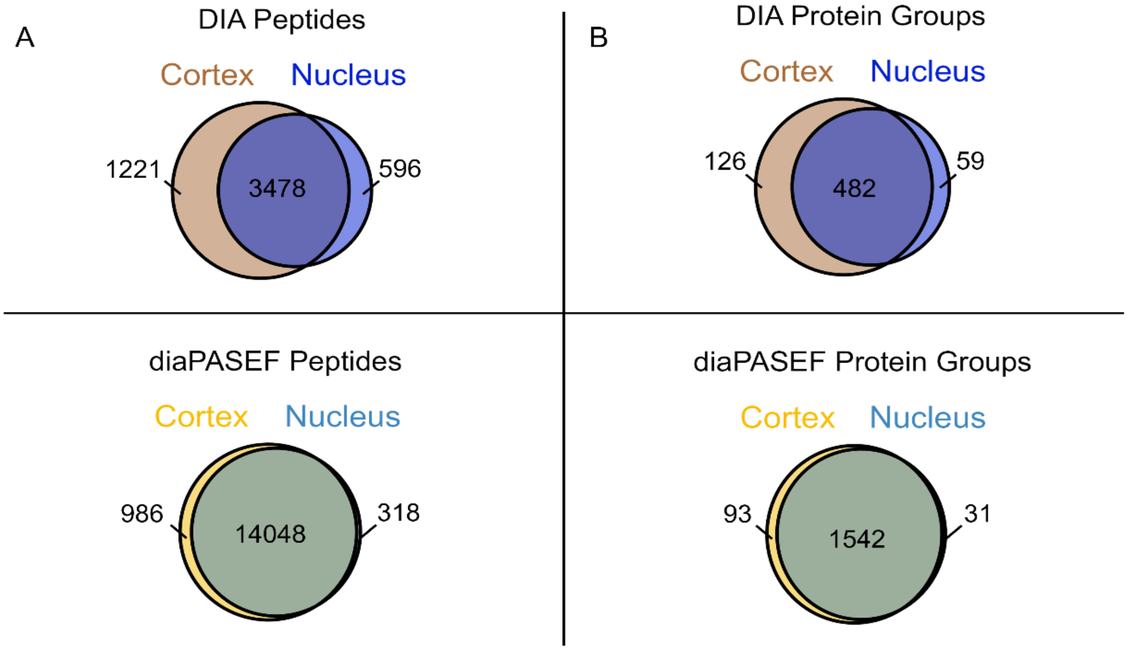

Supplemental Figure 3. Venn diagrams showing the overlap between A) peptides and **B)** protein groups between the DIA and diaPASEF data collected on the separated zebrafish lenses. All peptides and protein groups found in at least one biological replicate were compared, with duplicates removed.

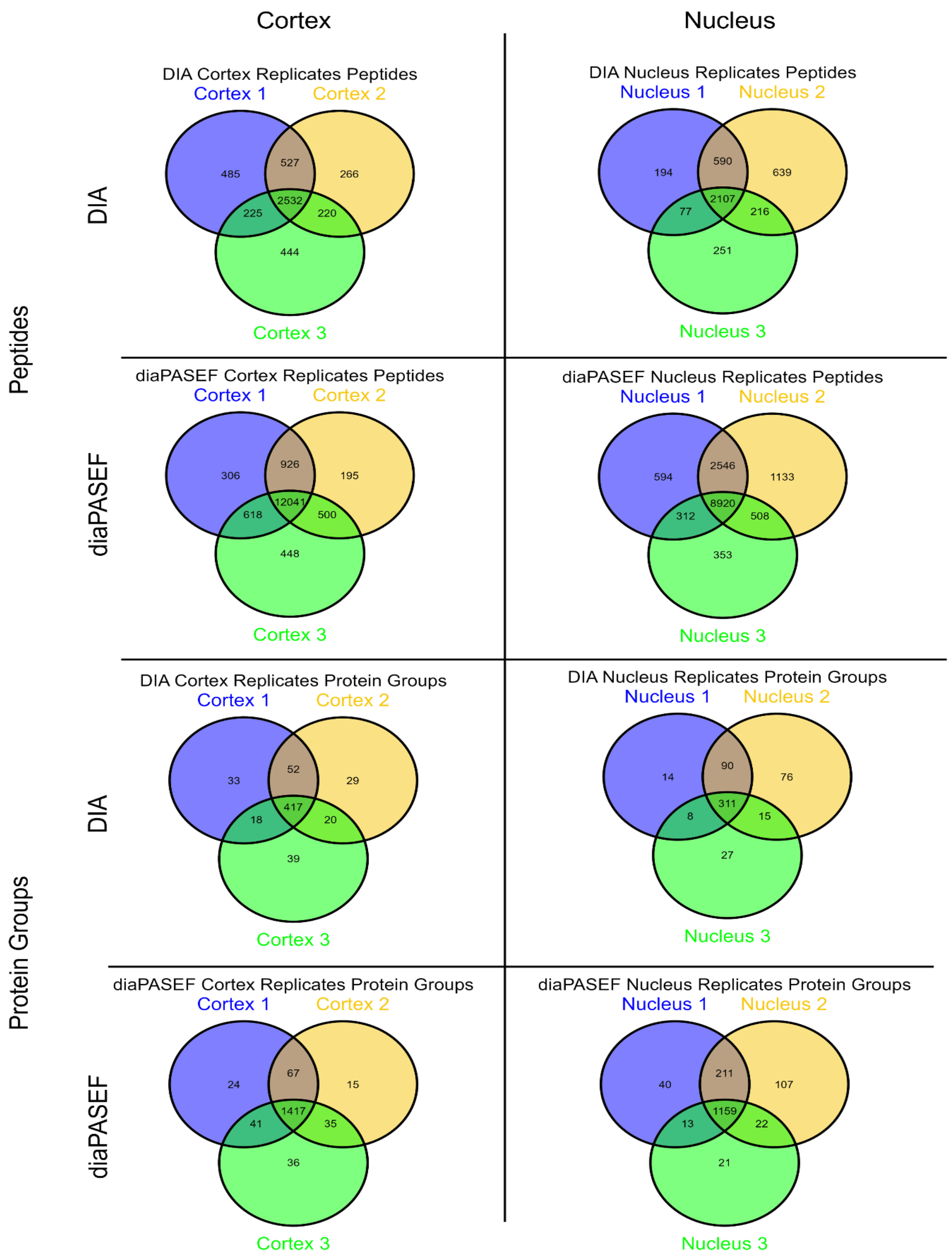

Supplemental Figure 4. Venn diagram showing the overlap between three biological replicates for zebrafish lens cortex and nucleus samples on the peptide and protein group level separated by data acquisition method.

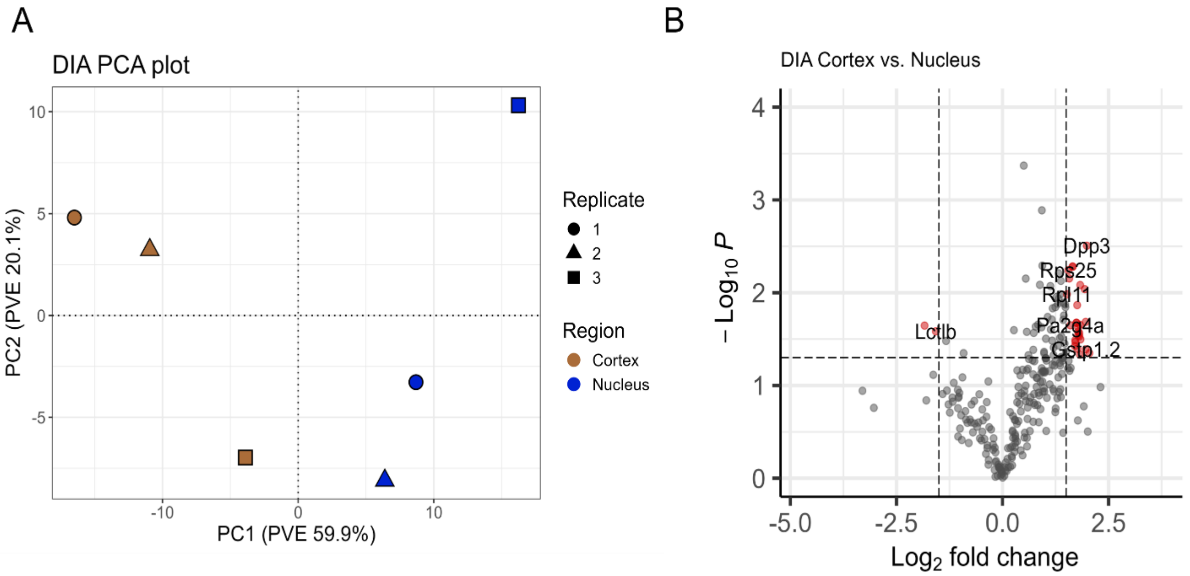

Supplemental Figure 5. Analysis results of orbitrap DIA data. A) PCA plot separated by replicate and region (n = 263). **B)** Volcano plot of cortex vs nucleus sample proteins. Significant protein groups are shown in red with cut-offs of $log_2FC > 1.5$ or $log_2FC < -1.5$ and p value < 0.05 as calculated from Welch's t-test.

Supplemental Table 1. Significant proteins (*p* value < 0.05 and log_2FC > 1.5 or log_2FC < -1.5) in the diaPASEF dataset organized by Log_2FC (n = 35).

Gene Group	Protein Group	Protein Description	*p* value	Log_2FC
rps8	P62247	Small ribosomal subunit protein eS8	0.003909041	2.481897098
rpl8	Q6P0V6	Large ribosomal subunit protein uL2	0.010171792	2.25857545
rpl27a	Q6P2M0	Large ribosomal subunit protein uL15	0.008776574	2.153349959
rpl36	Q6Q415	Large ribosomal subunit protein eL36	0.007524512	2.124129742
nat16	E7FET0	N-acetyltransferase 16	0.031498825	2.070295384
rpl34	Q7ZWJ7	Large ribosomal subunit protein eL34	0.003563581	2.069496022
rpl10	Q7ZV96	Large ribosomal subunit protein uL16	0.013188797	2.036976369
faua;faub	F1RA98;Q6PC01	FAU ubiquitin-like and ribosomal protein S30 fusion b	0.006760121	2.019043612
rpl21	Q6IQQ0	60S ribosomal protein L21	0.011212962	2.002765545
rbbp9	Q1MT41	Hydrolase RBBP9	0.006132191	1.925186015
rpl26	Q7SXA1	Large ribosomal subunit protein uL24	0.009230474	1.924398846
rpl13	Q90Z10	Large ribosomal subunit protein eL13	0.019915798	1.91269277
rps26l	Q6PBZ2	40S ribosomal protein S26	0.011059105	1.898059165
rps24	A0A8M2B343	40S ribosomal protein S24	0.012945126	1.77181796
rpl28	F1QFV6	Large ribosomal subunit protein eL28	0.008572089	1.767039669
rpl31	Q24JV3	Large ribosomal subunit protein eL31	0.007176367	1.765724655
zgc:171772	A7YY10	60S ribosomal protein L37a	0.013683676	1.709087001
rpl7a	Q6PBZ1	60S ribosomal protein L7a	0.000688221	1.638893239
gstp1.2	Q9DDU5	Glutathione S-transferase	0.030139252	1.555262716
si:dkey-164f24.2	A0A286Y8V3	Si:dkey-164f24.2	0.018064922	1.533536117
cav1	Q6YLH9	Caveolin	0.033681486	-1.502378174
atp2b1a	A0A8M9Q5T9	Calcium-transporting ATPase	0.005726974	-1.521883362
lctlb	A0A286Y8V0	Lactase-like b	0.033393737	-1.606722692
slc20a1b	Q6PFM1	Sodium-dependent phosphate transporter 1-B	0.015976032	-1.632235297
tmem47	Q6PFT6	Transmembrane protein 47	0.013242156	-1.665511898
rac1b	Q29RC5	Ras-related C3 botulinum toxin substrate 1	0.033802902	-1.688233208
misp	A0A0R4IHS7	Mitotic interactor and substrate of PLK1 isoform X1	0.034788062	-1.733434488
enpp6	Q5BKW7	Glycerophosphocholine cholinephosphodiesterase ENPP6	0.003057799	-1.791046449
zmp:0000000625	A0A8M1RIF4	Cadherin-2-like	0.021237841	-1.833211042
si:dkey-57a22.15	B0UYP0	Gamma-crystallin A	0.036082097	-1.913493079
lim2.5	Q5BKW4	Lens intrinsic membrane protein 2.5	0.001665345	-1.967881439
aqp0a	Q6DEI6	Aquaporin-0a	0.009523115	-2.040109434
gja8b	Q503J6	Gap junction protein	0.024193848	-2.058087606
aqp0b	Q4ZJI3	Aquaporin-0b	0.006478904	-2.239139865
lim2.3	Q6DGY5	Lens intrinsic membrane protein 2.3	0.021196919	-2.782351794

Supplemental Table 2. Significant proteins (*p* value < 0.05 and $log_2FC > 1.5$ or $log_2FC < -1.5$) in the orbitrap DIA dataset organized by Log_2FC (n = 29).

Gene Group	Protein Group	Protein Description	*p* value	Log_2FC
glo1	Q6P696	Lactoylglutathione lyase	0.044777548	2.046854321
dpp3	Q6DI20	Dipeptidyl peptidase 3	0.003115512	1.986749978
gstp1.2	Q9DDU5	Glutathione S-transferase	0.04077937	1.960622523
si:dkey-164f24.2	A0A286Y8V3	Si:dkey-164f24.2	0.020644478	1.959728754
rpl13	Q90Z10	Large ribosomal subunit protein eL13	0.009156845	1.937545666
rpl24	Q8JGR4	Large ribosomal subunit protein eL24	0.031828165	1.838986254
rps5	Q6PC80	Small ribosomal subunit protein uS7	0.008268908	1.830822172
eif3a	Q6PCR7	Eukaryotic translation initiation factor 3 subunit A	0.022164875	1.825577397
rpl23	Q6PC14	Large ribosomal subunit protein uL14	0.046451327	1.823766909
eno1a	A0A2R8Q1X2	Phosphopyruvate hydratase	0.029408901	1.812812676
rpl14	Q6DRN7	Large ribosomal subunit protein eL14	0.027148089	1.810202976
rps4x	Q642H9	Small ribosomal subunit protein eS4	0.026320039	1.781929636
rps14	Q6PBW3	Small ribosomal subunit protein uS11	0.023545001	1.780290144
aldoaa	Q803Q7	Fructose-bisphosphate aldolase	0.028904612	1.779777615
ppiab	Q6PC53	Peptidyl-prolyl cis-trans isomerase	0.040207925	1.766863159
rps13	Q6IMW6	Small ribosomal subunit protein uS15	0.013603921	1.760065774
rps23	A8KB78	Small ribosomal subunit protein uS12	0.021091342	1.7441086
phgdh	F1QEY8	D-3-phosphoglycerate dehydrogenase	0.021081516	1.738861491
hspb1	A4VAK4	Heat shock protein beta-1	0.033953265	1.716295077
rps18	Q8JGS9	Small ribosomal subunit protein uS13	0.032440526	1.714271327
mapk1	Q7ZW72	Mitogen-activated protein kinase	0.03757024	1.705442809
ass1	Q66I24	Argininosuccinate synthase	0.005244634	1.655897175
rpl7	Q6Q417	Large ribosomal subunit protein uL30	0.005221744	1.649584684
pa2g4a	Q8AW82	Novel protein similar to human proliferation-associated 2G4 protein (PA2G4)	0.022440603	1.597685365
rps16	Q1LWH1	40S ribosomal protein S16	0.007020197	1.572988422
rps25	Q6PBI5	Small ribosomal subunit protein eS25	0.005709555	1.549377184
rpl11	Q6IQI6	Large ribosomal subunit protein uL5	0.010395899	1.518867893
lctlb	A0A286Y8V0	Lactase-like b	0.026071736	-1.573217191
aqp0b	Q4ZJI3	Aquaporin-0b	0.022614919	-1.837531594

Supplemental Table 3. Overrepresentation analysis of statistically significant cortical orbitrap DIA proteins (p value < 0.05 and log_2FC > 1.5).

GO Term	Annotation	# in Set	Fold Enrichment	FDR
**GO Biological Process**				
GO:0042254	Ribosome biogenesis	6	11.19	6.22E-03
GO:0006412	Translation	13	5.1	6.81E-04
**GO Molecular Function**				
GO:0019843	rRNA binding	5	23.68	4.12E-04
GO:0003735	Structural constituent of ribosome	14	11.34	8.87E-10
**GO Cellular Component**				
GO:0022627	Cytosolic small ribosomal subunit	7	14.36	2.16E-05
GO:0022625	Cytosolic large ribosomal subunit	6	9.01	1.96E-03
GO:0016020	Membrane	0	< 0.01	0.0302
